# An In-vitro Comparison of the Shear Bond Strength of Three Different Composite Materials with Three Different Etchants for the Bonding of Orthodontic Brackets

**DOI:** 10.7759/cureus.4944

**Published:** 2019-06-19

**Authors:** Sharat Megh, VV Rao, MS Minor Babu, Punithavathy R., Martha Satyam, Akhil Pallepati, Raparla Mythraiye, Chandrika Paravada

**Affiliations:** 1 Pedodontics and Preventive Dentistry, Lenora Institute of Dental Sciences, Rajahmundry, IND; 2 Pedodontics and Preventive Dentistry, Lenora Institute of Dental Sciences, Rajahmundry , IND; 3 Public Health Dentistry, Lenora Institute of Dental Sciences, Rajahmundry, IND

**Keywords:** shear bond strength, orthodontic brackets, composites, etchants

## Abstract

Introduction

Esthetics, being the major concern of today’s treatments, has led to numerous innovations, including composites, for different treatment options. Esthetic orthodontics requires the use of composites for bonding orthodontic brackets to the teeth.

Aims

To identify which combination of composites has the highest shear bond strength at the tooth-bracket interface.

Materials and methods

Three different composite kits were selected for each group (n=42) and were further divided into three subgroups (n=14), where the bonding agents and/or primer were interchanged to find the best combination.

Results

Sub-group B2 (Orthofix + Eazetch + Universal Bond) showed the highest shear bond strength (10.74 ± 3.45 MPa), which was highly significant at *p* =/<0.0001.

Conclusion

The highest shear bond strength was found with the combination of 37% phosphoric acid (Eazetch), GC Universal Bond (GC Corporation, Tokyo, Japan), and Orthofix composite material (Anabond Stedman, Chennai, India). As this study is an in-vitro study, we need longitudinal in-vivo studies to establish the best combination for the bonding of orthodontic brackets.

## Introduction

Composites work wonders in young children and adolescents where esthetics and social acceptability are prime concerns. They have become the most accepted and widely used material by dentists performing orthodontic treatments all over the world. Bonding systems for composites have been undergoing continuous innovation. Bonding is a technique-sensitive procedure, where moisture is one of the common causes of bond failure at the tooth-adhesive interface and/or adhesive-bracket interface [1].

Orthodontic brackets in the oral cavity are subjected to several dislodgement forces and are influenced by factors such as the surface area of the tooth, conditioning procedures and materials used, composite material used, design of the bracket base, surface treatment of the bracket base, and the protocol followed during bonding to the tooth [2]. The changes occurring in the enamel microstructure also influence the bonding failure at the enamel-composite interface [3].

A few composite materials have been accepted in the dental practice for restorations as well as orthodontic bonding, and there is continuous, ongoing research to better the material. The aim is to achieve a material that can bond easily with fewer steps and can avoid bonding failures. Numerous studies have been conducted considering different materials under various conditions for orthodontic shear bond strength. The consideration of interchanging the different adhesive system kits was lacking.

## Materials and methods

The study was conducted in the department of pedodontics and preventive dentistry, Lenora Institute of Dental Sciences. Premolars extracted for orthodontic purposes were collected and a shear bond strength test was performed with an Instron Universal Testing machine (Instron, MA, US) at Gitam’s College of Engineering, Visakhapatnam.

A total of 126 premolars were collected and immediately stored in a saline solution. The premolars were cleaned using a brush and disinfected with 0.5% sodium hypochlorite solution. Following disinfection, the teeth were stored in saline solution at room temperature.

The premolars, once prepared, were divided into three major groups based on the composite material used:

1. Group A - 3M ESPE Filtek Z250 XT, Nano Hybrid Universal Adhesive (n=42)

2. Group B - Orthofix - Light cure Orthodontic Bonding System (n=42)

3. Group C - GC - Solare Sculpt - Nano Hybrid Universal Composite (n=42)

The three major groups were each further subdivided into three sub-groups based on the etchant/primer used:

i. Sub-group A1 - 3M Single Bond Universal Adhesive (n=14)

ii. Sub-group A2 - Eazetch + GC Universal Bond (n=14)

iii. Sub-group A3 - Eazetch + Orthofix Primer (n=14)

iv. Sub-group B1 - 3M Single Bond Universal Adhesive (n=14)

v. Sub-group B2­ - Eazetch + GC Universal Bond (n=14)

vi. Sub-group B3 - Eazetch + Orthofix Primer (n=14)

vii. Sub-group C1 - 3M Single Bond Universal Adhesive (n=14)

viii. Sub-group C2 - Eazetch + GC Universal Bond (n=14)

ix. Sub-group C3 - Eazetch + Orthofix Primer (n=14)

This was done to identify which combination of composite - etchant/primer has a better bond strength at the bracket-tooth interface. After bonding with orthodontic brackets, the teeth were stored in saline solution, at room temperature, for seven days. After seven days, the teeth were subjected to a shear bond strength test with a universal testing machine and the values were recorded.

The teeth were then viewed under a stereomicroscope with 10x and 40x magnification to check for adhesive remnants on the buccal surface of the teeth and the findings were recorded with the adhesive remnant index.

## Results

The shear bond strength test was carried out using an Instron Universal testing machine, and the adhesive remnants were viewed under a stereomicroscope, with 10x and 40x magnification, to record the adhesive remnants. The results were subjected to a one-way analysis of variance (ANOVA) statistical analysis.

The results show the shear bond strength of Group A, ranging from 3.31 ± 1.44 MPa to 8.75 ± 5.24 MPa. Sub-group A2 showed the highest shear bond strength, which was found to be highly significant at p = 0.004. A post-hoc comparison between the sub-groups showed a highly significant p-value between sub-groups A2 and A3, in favor of A2.

The results for group B range from 4.03 ± 1.63 MPa to 10.74 ± 3.45 MPa. The highest shear bond strength value was recorded with sub-group B2 at 10.74 ± 3.45 MPa, which was highly significant at p =/< 0.0001. The post-hoc comparison of sub-groups showed a highly significant p-value between sub-groups B1, B2, and B3, in favor of sub-group B2, which used the same etchant/primer system as sub-group A2.

The results for group C range from 1.84 ± 1.12 MPa to 7.78 ± 4.19 MPa. The highest shear bond strength value was recorded with sub-group C2 at 7.78 ± 4.19 MPa, which was highly significant at p =/< 0.0001. The post-hoc comparison of sub-groups showed a high significance and a p-value between sub-groups C1, C2, and C3, in favor of sub-group C2, which used the same etchant/primer system as sub-groups A2 and B2.

The overall nine sub-groups comparison showed a highly significant p-value at <0.0001 in favor of sub-group B2. Along with the shear bond strength tests, adhesive remnants were also recorded based on the amount of adhesive that remained on the tooth surface after debonding with 10x magnification and 40x magnification and the values were analyzed with the one-way ANOVA statistical test. These findings are shown in Tables 1-6.

**Table 1:** Nine-groups inter-comparison ANOVA: analysis of variance

Group	Shear bond strength (Mean ± SD)	One-way ANOVA (F) value	p-value
Group A1	6.38±4.25	11.507	<0.0001**
Group A2	8.75±5.24		
Group A3	3.31±1.44		
Group B1	5.99±3.49		
Group B2	10.74±3.45		
Group B3	4.03±1.63		
Group C1	1.84±1.12		
Group C2	7.78±4.19		
Group C3	3.11±1.39		

**Table 2:** Nine-groups post-hoc inter-comparison

Group	Group	p-value
Group A1	Group A2	0.601
	Group A3	0.244
	Group B1	1.000
	Group B2	0.016*
	Group B3	0.606
	Group C1	0.01**
	Group C2	0.968
	Group C3	0.173
Group A2	Group A3	0.001**
	Group B1	0.385
	Group B2	0.791
	Group B3	0.006**
	Group C1	<0.0001**
	Group C2	0.997
	Group C3	<0.0001**
Group A3	Group B1	0.429
	Group B2	<0.0001**
	Group B3	1.000
	Group C1	0.955
	Group C2	0.012*
	Group C3	1.000
Group B1	Group B2	0.006**
	Group B3	0.809
	Group C1	0.027*
	Group C2	0.872
	Group C3	0.328
Group B2	Group B3	<0.0001**
	Group C1	<0.0001**
	Group C2	0.291
	Group C3	<0.0001**
Group B3	Group C1	0.693
	Group C2	0.068
	Group C3	0.998
Group C1	Group C2	<0.0001**
	Group C3	0.982
Group C2	Group C3	0.007*

**Table 3:** Nine-groups inter-comparison at 10x magnification ARI: adhesive remnant index; ANOVA: analysis of variance

Group	ARI score (Mean ± SD)	One-way ANOVA (F) value	p-value
Group A1	0.85±0.94	3.57	0.001**
Group A2	1.21±1.05		
Group A3	1.07±0.82		
Group B1	1.00±1.07		
Group B2	1.35±0.63		
Group B3	0.50±0.51		
Group C1	1.78±0.80		
Group C2	1.35±0.49		
Group C3	1.28±1.57		

**Table 4:** Nine-groups post-hoc inter-comparison at 10x magnification

Group	Group	p-value
Group A1	Group A2	0.924
	Group A3	0.997
	Group B1	1.000
	Group B2	0.652
	Group B3	0.924
	Group C1	0.023*
	Group C2	0.652
	Group C3	0.813
Group A2	Group A3	1.000
	Group B1	0.997
	Group B2	1.000
	Group B3	0.183
	Group C1	0.475
	Group C2	1.000
	Group C3	1.000
Group A3	Group B1	1
	Group B2	0.979
	Group B3	0.473
	Group C1	0.183
	Group C2	0.979
	Group C3	0.997
Group B1	Group B2	0.924
	Group B3	0.652
	Group C1	0.099
	Group C2	0.924
	Group C3	0.979
Group B2	Group B3	0.05*
	Group C1	0.813
	Group C2	1.000
	Group C3	1.000
Group B3	Group C1	<0.0001**
	Group C2	0.05*
	Group C3	0.099
Group C1	Group C2	0.813
	Group C3	0.652
Group C2	Group C3	1.000

**Table 5:** Nine-groups inter-comparison at 40x magnification ARI: adhesive remnant index; ANOVA: analysis of variance

Group	ARI score (Mean ± SD)	One-way ANOVA (F) value	p-value
Group A1	1.21±0.89	3.63	0.001**
Group A2	1.42±0.93		
Group A3	1.35±0.84		
Group B1	1.07±0.47		
Group B2	1.71±0.72		
Group B3	0.85±0.36		
Group C1	2.07±0.73		
Group C2	1.64±0.63		
Group C3	1.57±0.64		

**Table 6:** Nine-groups post-hoc inter-comparison at 40x magnification

Group	Group	p-value
Group A1	Group A2	0.997
	Group A3	1.000
	Group B1	1.000
	Group B2	0.651
	Group B3	0.924
	Group C1	0.04*
	Group C2	0.812
	Group C3	0.924
Group A2	Group A3	1.000
	Group B1	0.924
	Group B2	0.979
	Group B3	0.472
	Group C1	0.308
	Group C2	0.997
	Group C3	1.000
Group A3	Group B1	0.979
	Group B2	0.924
	Group B3	0.651
	Group C1	0.183
	Group C2	0.979
	Group C3	0.997
Group B1	Group B2	0.308
	Group B3	0.997
	Group C1	0.01**
	Group C2	0.472
	Group C3	0.651
Group B2	Group B3	0.049*
	Group C1	0.929
	Group C2	1.000
	Group C3	1.000
Group B3	Group C1	0.001**
	Group C2	0.099
	Group C3	0.183
Group C1	Group C2	0.812
	Group C3	0.651
Group C2	Group C3	1.000

In the present study, results revealed that most of the adhesive remnants were seen in sub-group C1, which showed a higher bonding at the tooth-adhesive interface, followed closely by sub-group B2, which also had higher shear strength values.

The higher values of sub-group C1 with adhesive remnants could be related to the high bonding failure at the bracket-adhesive interface thus showing low shear bond strength in comparison to sub-group B2 and high adhesive remnants due to bond failure at the tooth-adhesive interface.

## Discussion

In recent years, esthetics and self-consciousness regarding teeth alignment have increased multifold. The acceptance of esthetic treatments led to the incorporation of composites as the adhesive for bonding brackets to the young permanent teeth.

The bond failure of orthodontic brackets on teeth occurs due to an improper bonding technique or poor material bond strength. The present study was conducted with established materials used for orthodontic bracket bonding. In this study, a combination of the materials was taken into consideration in the quest to find an improved combination of etchant-primer/bonding agent-composite.

Pediatric dentistry has seen the utilization of composite resin with a wide variety of uses, for example, pit and fissure sealants and the bonding of orthodontic brackets. The establishment of adhesive and restorative dentistry was laid by Michael Buonocore in 1955 with the idea of "Acid Etch Technique." The optimum results of acid etching were seen with 37% phosphoric acid for 15 s. The disadvantages of acid etching include the expulsion of a surface layer of enamel, variability in etch depth, contamination of the etched surface with water or oil, and insufficient washing or drying influencing the bond strength adversely [1,4].

Composite resin is the most esthetic material available today with predominant superior mechanical properties. They require bonding agents since they are normally hydrophobic and, consequently, don't stick well to teeth. Composites demonstrate a higher mean shear bond strength in permanent teeth while there is a significantly lower mean shear bond strength in primary teeth. Bordin-Aykroyd et al. proposed that the shear bond strength of dentin adhesive relies upon calcium level or the total area of strong dentin available [5]. The increased frequency of the cohesive failures, as well as the decreased frequency of adhesive, dictated that failures occurred within the composite resin rather than at the composite-bracket base interface or composite-specimen surface interface [6].

Based on the frequent bonding failures of orthodontic brackets to young permanent teeth, the present study was conducted on 126 premolars, extracted for therapeutic purposes and stored in saline solution. Cleaning and disinfection were done using a 0.5% sodium hypochlorite solution and a brush. Post cleaning and disinfection, the teeth were again stored in saline solution at room temperature. Care was taken to identify flawless teeth and ensure that all premolars were young and permanent and extracted for therapeutic purposes only.

The teeth were air-dried and embedded into acrylic blocks measuring 2 cm x 1 cm in dimension. The acrylic blocks were trimmed, cleaned, and dried. The teeth were also cleaned, dried, and polished with pumice before the procedure was started.

The teeth were then divided into three major groups (n=42) based on the composite material used. Each major group was further subdivided into three subgroups (n=14) based on the etchant and primer/bonding agents used.

Orthodontic brackets were bonded to the buccal surface of the premolars in accordance with the parameters set for each group and sub-group. The samples, once ready, were stored in saline solution for seven days. After seven days, the samples were taken to Gitam’s Institute of Engineering for testing the shear bond strength, using the Instron Universal testing machine, of the combination of materials used. After debonding, the teeth were viewed at 10x and 40x magnifications, under a stereomicroscope, to assess the adhesive remnants on the buccal surface of the premolars.

The results of the present study show that the shear bond strength was highest when the tooth surface was etched with 37% phosphoric acid (Eazetch, Anabond), followed by GC Universal Bond (primer/bonding agent) with Orthofix Light cure Orthodontic Bonding System in sub-group B2. This group also showed the second highest adhesive remnant scores, proving to be the best combination for orthodontic bracket bonding on teeth.

## Conclusions

In the present study, the highest shear bond strength was found with the combination of 37% phosphoric acid, GC Universal Bond, and Orthofix composite material. As this is an in-vitro study, we need longitudinal in-vivo studies to establish the best material for the bonding of orthodontic brackets.
